# The Performance of Operations

**Published:** 1915-04-10

**Authors:** B. Burford Rawlings


### THE EVOLUTION OF THE MODERN HOSPITAL.*

### VI.—The Performance of Operations.

By B. BURFORD RAWLINGS.

The cases following were mentioned to the writer by two gentlemen—one a layman, the other a surgeon, both eminent in the hospital world. (1) Two Canadian surgeons attended by invitation certain surgical practice at a hospital in London. They went away before the operation was completed, preferring to take no further part, even as spectators. In conversation afterwards, they expressed amazement at what had been attempted, and said it went beyond anything that would be permitted in Canada or the United States. (2) A member of the staff of a hospital, also in London, roundly accused a colleague of performing, without the friends' permission, an uncalled-for and in his opinion unjustifiable operation upon a patient who died from its effects. As a personal acquaintance of the patient he threatened proceedings, but was dissuaded, in the interest of the staff, from making the facts public.

To some minds even the labours of the pathologist are not tolerable, and a knowledge that *post-mortem* examination and mutilation of patients' bodies take place is inimical to the financial welfare of the hospitals. The experience of St. Bartholomew's affords fresh proof of the unwillingness of the ordinary donor to help any undertaking not of direct and immediate benefit to the patients. The object appealed for was the erection of buildings for pathological purposes, and the ensuing disappointment enforces a lesson long ago learned by experienced financiers. Dependence upon voluntary aid demands constant concession to sensibilities, and even to prejudices. Times out of number it has been shown that an appeal founded upon the scientific value of a hospital is apt to render disservice to the cause advocated. So far from showing willingness that his subscription shall be shared by the school, the ordinary subscriber thinks that the cost of medical education should be borne by the profession and the student. He desires to assist in the relief of suffering present and personal; the prospect of rendering society at large a future and problematical service has for him no attraction.

Seeing that the patients in a hospital ward have no protection but the character and reputation of the individual members of the staff in whose charge they are placed, confidence must suffer when it is understood how large a portion of the treatment is initiated and carried out by substitutes, and that patients and friends are given no facilities for making complaints. Even when matters affecting the conduct of hospital officers become the subject of public inquiry, independent medical assistance to establish the truth is rarely forthcoming. When an instrument had been left by accident inside a patient's body after operation, and death ensued, it was suggested that hospital patients and their friends must find consolation for occasional mishaps in the fact that they enjoy without payment the services of distinguished and busy practitioners. This view received almost immediate endorsement in another similar case, attended happily with less lamentable results, which found its way to the Law Courts, where the jury, while returning a verdict for the plaintiff, refused damages on the ground that the services of the defendant were gratuitous, and not until the judge had interposed was the plaintiff awarded a sum of £25, and "that only in consideration of her suffering."

The custom in some hospitals to perform serious operations upon patients without the consent, or even the knowledge, of friends has been frequently animadverted upon. Some time ago the board of Middlesex Hospital declared against this practice and, whenever possible, consent is to be obtained in writing, a welcome concession to the feelings of patients and their friends, which, if adopted at all hospitals, would dispose of a substantial grievance.

In a recent action, "Hillyer *v.* the Governors of St. Bartholomew's Hospital," the plaintiff, a medical practitioner, sued for compensation for injuries inflicted, so he alleged, by careless handling while undergoing examination under an anæsthetic. The facts do not appear to have been disputed, but the defendants denied liability upon the pleas that the examination was made "gratuitously," and that there was no relation of master and servant existing between themselves and those engaged in conducting the examination which made them responsible in law. Could any exposure of the existing relations of staffs and hospitals and the impotence of the management be more complete or more condemnatory? In the Court of first instance the plea prevailed immediately; the Judge withdrew the case from the jury. Upon appeal the Master of the Rolls, with Lords Justices Farwell and Kennedy, took time to consider their judgment, and ultimately upheld the decision of the Court below. If it be the fact, as seems to have been decided more than once, that medical and surgical work undertaken without fee does not involve legal responsibility for due performance, the position of a hospital patient in relation to the practitioner is that of an outlaw, while the old legal maxim that every wrong has its remedy receives a fresh and startling refutation.

* Previous articles: February 20, March 6, 13, 20, 27, and April 3.

